# Effects of school myopia management measures on myopia onset and progression among Chinese primary school students

**DOI:** 10.1186/s12889-023-16719-z

**Published:** 2023-09-19

**Authors:** Jiao- jiao Shi, Yu-jie Wang, Ping-ping Lyu, Jing-wen Hu, Xiao-sa Wen, Hui-jing Shi

**Affiliations:** 1 Department Maternal Child & Adolescent Health, School of Public Health, Fudan University, 138 Yixueyuan Road, Shanghai, 200032 China; 2 Department of Immunizations, Minhang Centers for Disease Control and Prevention, Shanghai, 201100 China

**Keywords:** Myopia, School management measures, Children, Multilevel analysis

## Abstract

**Background:**

Schools play an organizational role in managing myopia-related behavioral habits among students. We evaluated the effects of school myopia management measures on myopia onset and progression in a school-based prospective 1-year observational study.

**Methods:**

In total, 8319 children from 26 elementary schools were included. Online questionnaire completed by a parent, in which school myopia management experience including outdoor activities in recess or physical education class, teachers’ supervision, and teaching facilities. Variables were defined as implemented well or poorly, according to the Comprehensive Plan to Prevent Myopia among Children and Teenagers. Children underwent ophthalmic examinations, and the incidence and progression of myopia from 2019 to 2020 were estimated. Multilevel logistic regression models were constructed to analyze the association between school management measures and myopia development in 8,9 years and 10,11 years students.

**Results:**

From 2019 to 2020, the incidence of myopia among primary school students was 36.49%; the mean difference of spherical equivalent in myopic children was − 0.29 ± 1.22 diopters. The risk of incident myopia was reduced by 20% in 8,9 years participants with well-implemented class recess compared with those with poorly implemented class recess (adjusted odds ratio [aOR]: 0.80, *p* = 0.032). PE outdoor time was significantly associated with myopia incidence in 10,11 years students (aOR: 0.76, *p* = 0.043). Compared with poorly implemented reading and writing posture, desk and chair height, 10,11 participants with well-implemented desk and chair height were less likely to have rapid myopic progression (*p* = 0.029, *p* = 0.022).

**Conclusion:**

In Shanghai, children’s myopia is associated with better implementation of school myopia management measures. The present findings suggest that outdoor activities during class recess or PE class, providing suitable desks and chairs, and adequate instruction in reading and writing postures might protect against pathological eye growth. An age-specific myopia prevention and control programs in school is of primary importance.

## Background

Myopia is a serious public health issue in China and worldwide [1, 2]. China is among the countries with the highest prevalence of myopia globally, with the prevalence of 53.60% among children and adolescents, according to 2018 data released by the National Health Commission. Genetic and environmental factors (notably higher education level and limited outdoor time [3–5]) are associated with myopia onset and progression. Intensive education is a strong risk factor for myopia [6, 7]. However, how to prevent and control myopia incidence and development remains unclear according to present research evidence.

As an important part of social networks, schools play an organizational role in managing myopia-related behavioral habits among students. Time spent at school includes the best hours for outdoor activities, with the school day in China lasting from 8 a.m. to 5 p.m. Similar to the motivational effect of positive parental behaviors on children’s myopia [8], school teachers not only teach students about visual health knowledge directly but also influence their attitudes and behaviors to a large extent [9–11]. Since the Comprehensive Plan to Prevent Myopia among Children and Teenagers (CPPMCT) was released in 2018, schools in China have been required to instruct and monitor students’ myopia-related behavioral habits, ensure adequate outdoor activities for students, and providing a favorable visual environment [12, 13]. A large body of interventional studies has spotlighted the impact of single factors on myopia in children [14–16], including adding outdoor activity classes [3], installing overhead lighting systems in classrooms [17], and conducting myopic vision health training [10]. Although most schools have been implementing comprehensive myopia management measures under the guiding policy of the CPPMCT in China, there is still a lack of focused analysis of comprehensive school management measures in myopia. In addition, current school-based myopia studies are mainly based on single-level statistical analysis and ignore the hierarchical structure of school–class–student data as a community.

All of these findings suggest a need to systematically investigate the effects of school management measures on myopia incidence and progression, providing evidence to inform school myopia management policies. This study focused on the role of school management measures in myopia prevention under the CPPMCT policy.

## Materials and methods

### Study design and participants

This was a prospective 1-year observational study. In June 2020, multi-stage stratified whole-group random sampling, stratified by school and grade level, was conducted with classes as the sampling unit, all students within the class were surveyed. Overall, 26 primary schools were selected, with a total of 8587 children included in the questionnaire survey.

Information of the children was obtained using an online questionnaire answered by both the child and an adult proxy respondent (mother, 74.7%; father, 24.6%; others, 7.3%) familiar with the child’s health, supervised by the head teacher of the classes. In July 2020, 8319 elementary school students in grades 1–4 completed questionnaires. Meanwhile, students’ visual acuity data were obtained from the local Center for Disease Control and Prevention (CDC) refractive system and matched to the questionnaire data by name, date of birth, and sex. Among them, 5294 (63.64%) had refractive data both for 2019 and 2020. There was no difference in age or sex between the response (*n* = 5294) and nonresponse group (*n* = 3025).

This study conformed to the principles of the Declaration of Helsinki, and informed consent was signed by the participants’ parents. The Ethics Committee of the School of Public Health, Fudan University, approved this study (approval number IRB#2020–07-0836).

### Questionnaire on school management measures and environmental factors

According to the CPPMCT in the district of Shanghai, and referring to the relevant literature and normative guidelines [18, 19], we designed a student–parent questionnaire in 2019 to 2020. School myopia management experience contained 8 questions, which was completed by parents after they consulted with their child. These items illustrated the extent to which the school management measures have been implemented from the students' perspective. Each type of school myopia management experience was assessed using the following questions: “Did the children go outside the teaching building for 10-min class recess (a 10-min recess scheduled between every two classes in Chinese primary and middle schools) every weekday?” Answers were multiple choice: almost every recess, 3–4 times a day during recess, 1–2 times a day during recess. “Please fill in the actual time you spent outdoors during each PE class in weekday”. Answers were: about 40 min, about 30 min, about 20 min, about 10 min, hardly ever. “Please fill in the actual time you spent outdoors during each long recess (a 30-min recess scheduled between the second and third class in the morning) every weekday”. Answers were: about 30 min, about 25 min, about 20 min, about 15 min, hardly ever. Other school management measures items referred to teachers’ behaviors and school visual environment, questions included “Did teachers correct students’ incorrect reading and writing posture during class?” Answers were multiple choice: often, sometimes, occasionally, and never. “The teacher's blackboard writing in class should be neat and large enough to be clearly seen, did the teacher in class do that?” and “Did the teacher adjust the curtains, blackboard lighting or other lighting in the classroom according to the weather conditions so that the students could read the words on the blackboard or PowerPoint?” Answers were always: all teachers can do it, most teachers can do it, about half of the teachers do it, few teachers can do it, no teacher can do it. “Do you think your desk and chair are the right height for your height?” Answers were: the desk is so high that it is difficult to keep the distance between the eyes and the books 1 foot when reading and writing, the height of the desk is just right for my height, the desk are so low that I have to arch my back when reading and writing. “How about the response space on written test for each subject in school?” Answers were: very small, fair, sufficiently large. The content validity of all the items on the questionnaire was reviewed by expert panels. Based on previous literature, the following potentially relevant factors were collected in questionnaire as covariates: age (8, 9, 10, 11 years), sex (boy, girl), parental high myopia (none, at least one), average daily outdoor time after school (classified into < 2 h, ≥ 2 h) and school characteristic (public, private).

### Eye examination

The visual acuity data for this study were obtained from the District CDC Child and Adolescent Visual Acuity File, which was established in 2009. Refractive development is documented once a year for each child and adolescent between the ages of 4 and 18 years, and the medical institution under its jurisdiction (usually a community health service center) is responsible for refractive examinations. A physician and a qualified optometrist with operational training and unified guidance measured noncycloplegic refraction using a computerized optometer. Three consecutive readings were recorded and averaged as the final figure for each eye. If the difference between any two spherical lens measurements was greater than or equal to 0.50 diopters (D), additional measurements were taken and the average was calculated again. Studies have shown a high correlation between noncycloplegic and cycloplegic refraction tests in myopia screening, and China National Health Commission recommends the use of noncycloplegic for vision screening in schools [20]. In the present study, data from two refractive screenings from 2019 to 2020 were selected, including basic student information, visual acuity and noncycloplegic optometry.

### Variable definitions

Spherical equivalent (SE) equals sphere power plus 1/2 cylinder power. Myopia was defined as SE ≤  − 0.50D in the right eye and high myopia was defined as SE ≤  − 6.00D. Incident myopia was defined as myopia occurrence in children who were non-myopic at baseline. The difference in SE (ΔSE) between 2-year and baseline values for myopic children was calculated. Using *P*75 as the cut-off value, ΔSE values were divided into the rapid myopia progression group and the slow myopia progression group. Only children with full non-cycloplegia were included in the analysis of myopia onset and myopia progression.

According to the CPPMCT and associated standards of the China Education Ministry, we defined well-implemented school management measures as children always go outside during class recess, actual outdoor time > 30 min in each PE class, actual outdoor time > 20 min in each long recess, most teachers correct writing and reading postures in class, most teachers adjust classroom lighting to accommodate vision, desks and chairs fit students’ height, and response space on written test is sufficient; other responses were defined as poor implementation.

### Statistical analysis

The data were analyzed using IBM SPSS Statistics 23.0 and StataSE 15.0. *p* values < 0.05 were considered statistically significant. Continuous variables are presented as mean ± standard deviation, and categorical variables are presented as number and percentage. Differences between myopic and non-myopic students were determined using the chi-square test. Taking into account differences in the rate of onset and progression of myopia in children of different ages, two-level logistic regression models were used to assess the association between school management measures and myopia onset and myopic progression among 8- and 9-year-olds, 10- and 11-year-olds, respectively, after adjusting for confounding factors of baseline age, sex, and parental high myopia.

## Results

A total of 8587 primary school parent questionnaires were completed. After excluding schools with too few students (less than 20), duplicates, incomplete information, and students whose ages were out of range, 8319 children in 26 elementary schools were enrolled in the data analysis, including 4371 (52.54%) boys and 3948 (47.46%) girls. Students’ age ranged from 5 to 11 years, with an average age of 8.80 ± 1.16 years; 1224 (14.89%) children had one and more myopic parents; 1995(23.98%) children were outdoors for more than 2 h a day (Table 1).

**Table 1:** Demographic characteristics of schools and children

Characteristics of Children/Institution	N(%)
School Characteristic	
Public School	21(80.77)
Private School	5(19.23)
Children Sex	
Boy	4371(52.54)
Girl	3948(47.46)
Children Age/years	
8	2517(30.26)
9	2184(26.25)
10	1922(23.1)
11	1696(20.39)
Parental High Myopia	
None	6999(85.11)
One	1224(14.89)
Outdoor time after school/day	
< 2 h	6324(76.02)
≥ 2 h	1995(23.98)

### School myopia management measures

**Fig. 1:**
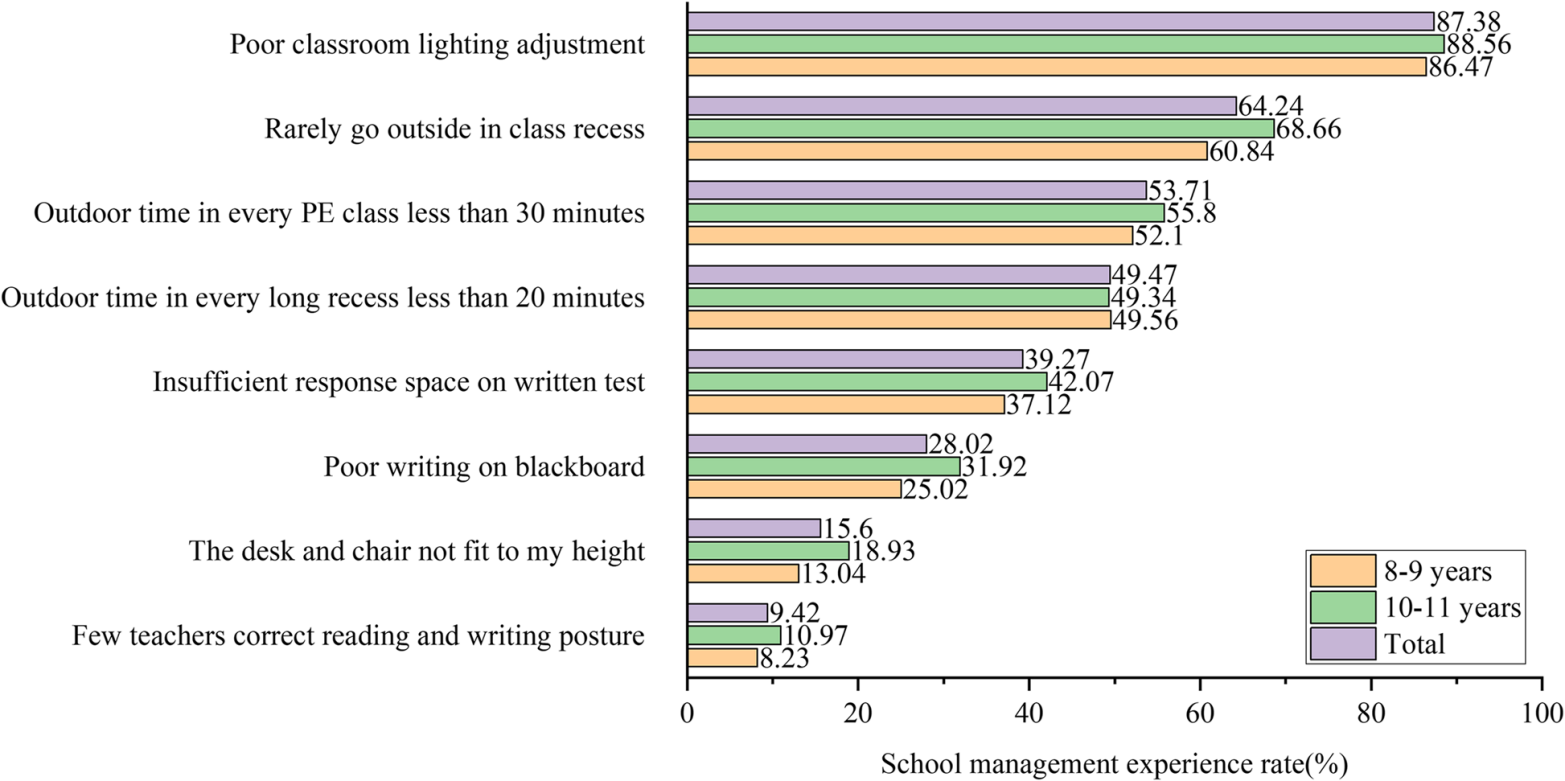
Proportion of school myopia management measures among elementary school students

Parents reported their child’s experience of school management measures at different ages, as depicted in Fig. 1. The most serious problem was that the classroom light environment was poor, reported by 87.38% (7269/8319) of students; this was followed by students rarely going outside in class recess (*n* = 5344, 64.24%) and outdoor time in each PE class less than 30 min (*n* = 4468, 53.71%). In addition, insufficient response space on written test was highlighted (*n* = 3267, 39.27%). Notably, in all school myopia management measures except for outdoor in every long recess less than 20 min, 10,11 years students had higher rates than 8,9 years students.

### Characteristics of incident myopia and rapid myopia progression

The prevalence of myopia among elementary school students in this study was 43.15% (2969/6879) in 2019 and 55.05% (3265/5931) in 2020; the mean SE was − 0.38 ± 1.17D in 2019 and 0.73 ± 1.41D. From 2019 to 2020, the incidence of myopia was 36.07% (1095/3001), and the mean myopia progression was − 0.29 ± 1.22D (2294).

10–11 years girls had higher myopia incidence (*p* = 0.004) and more rapid myopia progression than boys (*p* = 0.001). Participants with more than one high myopic parent were more likely to have myopia onset and more myopia progression (all *p* values < 0.01). 8,9 years students with well-implemented class recess outdoors had lower myopia incidence (*p* = 0.03). 10,11 years students with well-implemented PE outdoor time had lower myopia incidence (*p* = 0.046). 8,9 years students with well-implemented Long recess outdoors had more myopia progression (*p* = 0.046). A negative relationship was observed in 10,11 years participants between response space on written test (*p* = 0.016) and desk and chair height with myopia incidence (*p* = 0.022) (Table 2).

**Table 2:** Association between myopia and school management measures among elementary school students (Shanghai, 2019–2020)

Characteristics	8–9 years						10–11 years					
	Incident Myopia (*N* = 1948)			Rapid Myopia Progression (*N* = 1006)			Incident Myopia (*N* = 1013)			Rapid Myopia Progression (*N* = 1263)		
	n(%)	χ^2^	*p *value	n(%)	χ^2^	*p value*	n(%)	χ^2^	*p *value	n(%)	χ^2^	*p *value
Sex												
Boy	343(32.42)	0.25	0.618	337(20.93)	0.25	0.617	224(39.37)	8.19	**0.004**	277(23.14)	10.54	**0.001**
Girl	306(33.48)			299(21.68)			222(48.26)			323(29.07)		
Age/years												
8/10	322(30.73)	4.84	**0.028**	286(19.26)	7.18	**0.007**	257(43.05)	0.05	0.823	339(27.01)	1.47	0.225
9/11	327(35.39)			350(23.27)			189(43.75)			261(24.79)		
Parental High Myopia												
None	551(32.15)	6.63	**0.010**	502(19.83)	23.79	** < 0.001**	385(42.17)	8.06	**0.005**	484(24.51)	17.94	** < 0.001**
At least one	95(40.6)			128(30.33)			57(57)			109(35.97)		
Outdoor time after school/day												
< 2 h	493(33.49)	0.89	0.346	484(21.6)	0.55	0.460	333(43.76)	0.21	0.651	457(26.28)	0.29	0.588
≥ 2 h	156(31.2)			152(20.32)			113(42.16)			143(25.13)		
Class recess outdoor												
Poorly implemented	420(34.74)	4.73	**0.030**	405(22.17)	2.22	0.136	306(44.67)	1.47	0.225	415(26.45)	0.52	0.469
Well implemented	229(30.01)			231(19.88)			140(40.7)			185(25.03)		
PE outdoor time												
Poorly implemented	322(31.08)	3.31	0.069	343(22.07)	1.22	0.270	271(46.01)	3.99	**0.046**	356(27.2)	2.26	0.133
Well implemented	327(34.94)			293(20.42)			175(39.77)			244(24.42)		
Long recess outdoor												
Poorly implemented	292(30.77)	3.80	0.051	283(19.72)	3.99	**0.046**	223(43.55)	0.02	0.892	289(25.31)	0.56	0.454
Well implemented	357(34.9)			353(22.72)			223(43.13)			311(26.67)		
Writing on blackboard												
Poorly implemented	149(31.63)	0.46	0.499	172(23.69)	3.33	0.068	133(45.08)	0.51	0.475	201(28.19)	2.58	0.108
Well implemented	500(33.31)			464(20.5)			313(42.64)			399(25.02)		
Classroom lighting adjustment												
Poorly implemented	545(32.54)	0.7	0.402	547(21.48)	0.4	0.525	400(44.05)	1.58	0.208	534(26.25)	0.59	0.443
Well implemented	104(35.02)			89(20.14)			46(38.02)			66(24.09)		
Response space on written test												
Poorly implemented	239(32.34)	0.17	0.677	225(20.25)	1.11	0.292	168(38.98)	5.75	**0.016**	256(26.47)	0.2	0.657
Well implemented	410(33.25)			411(21.88)			278(46.49)			344(25.65)		
Reading and writing posture												
Poorly implemented	42(30.43)	0.41	0.521	48(21.52)	0.01	0.925	43(41.75)	0.12	0.731	77(30.31)	2.77	0.096
Well implemented	607(33.1)			588(21.26)			403(43.52)			523(25.46)		
Desk and chair height												
Poorly implemented	77(32.35)	0.04	0.845	80(21.28)	0	0.999	67(35.83)	5.25	**0.022**	119(27.67)	0.77	0.379
Well implemented	572(32.99)			556(21.28)			379(45.01)			481(25.61)		

### Effects of school myopia management measures on the development of myopia in elementary school students

The fitted null multilevel model with incident myopia and progression as response variables in 8,9 years and 10,11 years was statistically significant, and the level 2 residuals were statistically significant with* P* < 0.05. The results of multilevel analysis of school management measures in myopia are shown in Table 3. Well-implemented classes recess reduced the risk of incident myopia in 8,9 years students, with an adjusted OR 0.80 (*p* = 0.032). Well-implemented writing on blackboard reduced the risk of rapid myopia progression in 8,9 years students, with an adjusted OR 0.69 (*p* = 0.046). By contrast, PE outdoor time was significantly associated with 10,11 years students myopia incidence (OR: 0.76, *p* = 0.043). Furthermore, compared with poorly implemented reading and writing posture, desk and chair height, 10,11years students who had well-implemented reading and writing posture, desk and chair heights were less likely to have rapid progression of myopia, respectively (*p* = 0.029, *p* = 0.022).

**Table 3:** Two-level logistic analysis of school management measures in the incidence and progression of myopia (Shanghai, 2019–2020)

Characteristics	8–9 years				10–11 years			
	Incident Myopia (*N* = 1948)		Rapid Myopia Progression (*N* = 1006)		Incident Myopia (*N* = 1013)		Rapid Myopia Progression (*N* = 1263)	
	*OR*(95%*CI*)	*p *value	*OR*(95%*CI*)	*p *value	*OR*(95%*CI*)	*p *value	*OR*(95%*CI*)	*p *value
Sex								
Boy	1		1		1		1	
Girl	0.99(0.81,1.2)	0.892	0.81(0.6,1.1)	0.181	1.41(1.09,1.82)	0.01	1.21(0.94,1.56)	0.142
Age/years								
8/10	1		1		1		1	
9/11	1.17(0.96,1.42)	0.124	1.31(0.96,1.78)	0.094	1.06(0.82,1.37)	0.671	0.79(0.61,1.02)	0.067
Parental High Myopia								
None								
At least one	1.37(1.02,1.84)	**0.034**	1.79(1.25,2.57)	**0.001**	1.78(1.16,2.74)	**0.009**	1.67(1.19,2.35)	**0.003**
Outdoor time after school/day								
< 2 h	1		1		1		1	
≥ 2 h	0.91(0.72,1.14)	0.417	0.82(0.57,1.19)	0.293	0.96(0.72,1.29)	0.793	1.08(0.8,1.45)	0.634
Class recess outdoor								
Poorly implemented	1		1		1		1	
Well implemented	0.80(0.65,0.98)	**0.032**	0.86(0.63,1.19)	0.368	0.89(0.68,1.18)	0.435	0.99(0.74,1.31)	0.923
PE outdoor time								
Poorly implemented	1		1		1		1	
Well implemented	1.17(0.96,1.43)	0.130	0.9(0.66,1.23)	0.52	0.76(0.58,0.99)	**0.043**	0.87(0.67,1.13)	0.290
Long recess outdoor								
Poorly implemented	1		1		1		1	
Well implemented	1.21(0.99,1.48)	0.061	1.15(0.85,1.57)	0.363	1.01(0.78,1.31)	0.930	1.14(0.88,1.47)	0.330
Writing on blackboard								
Poorly implemented	1		1		1		1	
Well implemented	1.06(0.83,1.35)	0.643	0.69(0.48,0.99)	**0.046**	0.91(0.66,1.25)	0.552	0.97(0.72,1.29)	0.82
Classroom lighting adjustment								
Poorly implemented	1		1		1		1	
Well implemented	1.11(0.84,1.46)	0.467	0.79(0.49,1.27)	0.334	0.8(0.53,1.2)	0.28	0.79(0.52,1.19)	0.254
Response space on written test								
Poorly implemented	1		1		1		1	
Well implemented	0.96(0.78,1.19)	0.717	1.09(0.78,1.52)	0.62	1.28(0.97,1.7)	0.083	1.06(0.8,1.39)	0.688
Reading and writing posture								
Poorly implemented	1		1		1		1	
Well implemented	1.07(0.72,1.59)	0.741	1.32(0.75,2.34)	0.338	1.07(0.68,1.67)	0.777	0.65(0.44,0.96)	**0.029**
Desk and chair height								
Poorly implemented	1		1		1		1	
Well implemented	0.95(0.7,1.29)	0.761	0.92(0.59,1.43)	0.714	1.4(0.99,2)	0.061	0.69(0.5,0.95)	**0.022**
**Intercept**	0.28(0.1,0.8)	0.017	0.36(0.08,1.55)	0.17	0.34(0.11,1.08)	0.068	1.96(0.62,6.18)	0.252
**σ**^**2**^_**μ0**_	0.14(0.06,0.34)		0.16(0.06,0.42)		0.06(0.01,0.29)		0.49(0.22,1.06)	

## Discussion

This 1-year follow-up study revealed that the associations of different types of school myopia management measures with myopia incidence and progression may differ. Well-implemented outdoor activities significantly reduced the risk of myopia onset, and adequate instruction in reading and writing postures, appropriate student desk and chair height demonstrated less myopic progression in 10–11 years children. We constructed two-level models including school management measures to provide evidence regarding the effects of comprehensive school myopia management measures under the CPPMCT policy.

The current myopia prevalence in this survey was 43.15% in 2019 and the myopia incidence was 36.49% from 2019 to 2020, which is consistent with the prevalence according to the China Education Ministry [21]. The prevalence of myopia in primary school students in grades 4–6 in Ningbo is 61.5% [22]; 60.39% in Saybag District of Urumqi [23] and 33.6% in Chongqing [24]. The annual incidence of myopia was 20%–30% throughout Guangzhou in 2018 [25]. The yearly incidence of myopia increased from 7.8% in grades 1 and 2 to 25.3% in grades 5 and 6 in Anyang city [26]. The myopia prevalence in present study was much higher than the figures reported in many relevant studies. The great pressure of study among students in Shanghai in general may be one reason for our results. Noncycloplegic refraction used in our study may also overestimate myopia prevalence to some extent. Furthermore, the COVID-19 pandemic had led to an increase in myopia prevalence in China [27, 28].

This study showed that the risk of incident myopia was reduced by 20% in 8,9 years participants with well-implemented class recess (*p* = 0.032) while well-implemented PE outdoor time significantly reduced 10,11 years students’ incident myopia by 24% (*p* = 0.043). However, outdoor time after school and long recess outdoor were not significant in students’ myopia. We analyzed that this could be due to less natural light for outdoor activities after school and negligible variance in the data. Prior to our study, increasing outdoor time at school was known to be a protective factor against myopia [29]. In a school-based randomized controlled trial, the one-year myopia incidence declined after implementing outdoor time during recess [14].

On the one hand, frequent class recess outdoors or longer PE class outdoor time increased natural light exposure [3, 30], a process that was thought to be exerted by dopamine [31]. On the other hand, outdoors in recess or PE class effectively interrupt long duration of near work. Long continual near work was a cause of visual fatigue [32–34], which was deemed to be strongly associated with myopia [35, 36]. In China primary school, class recess and PE class are important part of children’s outdoor activities. Many students especially the upper grades may not go to outdoors spontaneously, but choose to stay in the classroom, or even continue to write and read [37]. As shown in our study, the proportion of students who rarely go outside between class recess was 60.8% in 8,9 years students, but 68.7% in 10,11 years students. There are also phenomena such as teachers occupying class recess and PE class [38, 39]. This may be partially contributed to the fact that the class recess was not significant in the occurrence of myopia in 10,11 years children. Indeed, high-density teaching buildings, high floors and increased time at home due to the COVID-19 [40], safety concerns of schools, especially in urban schools, making it difficult to promote outdoor activities. There is an urgent need to establish an outdoor-friendly campus environment, and implement age-specific measures. In addition, how the frequency and duration of outdoor activities are set in different grades deserves further study.

The present study found that well-guided reading and writing postures, well implemented writing on blackboard, well implemented desk and chair height reduced the risk of myopia development. The positive effect of teaching facilities on controlling the development of myopia were reported in previous studies. Zhao found that good classroom environmental health, consisting of classroom lighting and suitable desks and chairs, had a negative effect on myopia progression [41]. In He and You’s study, they found that the school desk height, and teacher's instruction on reading and writing posture were significantly associated with the progression of myopia [42, 43]. And the effect of lighting on myopia has been confirmed in many studies [17, 44, 45]. However, school management measures classroom lighting adjustment and response space on written test in present study did not reach the significance. We considered it may partly account for the different definition of lighting in our study and the overall low rates of school management implementation. Regarding the differences in the effectiveness of school-based management measures among 8–9, 10–11 years students, we believe that they were mainly caused by the differences in the development of myopia and children's myopia-related behaviors as one's age advances. An age-specific myopia prevention and control programs is of primary importance, encouraging proper guidance by teachers for all students and providing desks and chairs of appropriate height for upper grades may help enhancing the beneficial children’s behaviors.

Among the strengths of our study, we analyzed the impact of different school management practices on various myopia outcomes among 8–11 years students. Our study findings add to the evidence regarding the known protective effects of outdoor exposure and suggest that age-specific outdoor measures at school should be emphasized. Our study results suggest the positive effects of teaching facilities on myopia and the urgency of improving the visual environment and teacher supervision behaviors at the school level. Finally, data from previous studies have rarely considered a cluster structure, but we examined the organizational management role of the school in detail.

There are also some limitations in our study. First, the interval between the two vision screenings was too short that it was difficult to determine the association between myopia and school myopia management practices. Second, information on school management measures were obtained from student–parent questionnaires rather than from institutional surveys, although the former can reflect the actual implementation of management measures for individuals, the influence of personal habits and other environmental factors are difficult to avoid. Third, in our study, information was obtained using a questionnaire without objective and quantitative measurements, making it difficult to avoid recall bias. Finally, cycloplegic refraction was not used in the present study, which may lead to an overestimation of myopia. Nevertheless, further longitudinal studies with larger study populations are necessary to elucidate the relationship between school management, children’s behavior patterns, and myopia status overall.

## Conclusion

In summary, well-implemented school myopia management practices had a protective effect on student myopia in our study. Compared with other myopia management measures conducted in schools, our study clearly demonstrated that encouraging outdoor activities during recess or PE class and providing adequate instruction in reading and writing postures, good writing on blackboard, desks and chairs with suitable heights significantly delayed the development of myopia.

## Data Availability

The datasets used and/or analysed during the current study are available from the corresponding author on reasonable request.

## Abbreviations

CPPMCT: The Comprehensive Plan to Prevent Myopia among Children and Teenagers
aOR: Adjusted odds ratio
ICC: Correlation coefficient
CDC: Center for Disease Control and Prevention
PE: Physical education
SE: Spherical equivalent
PPT: Power Point
95% CI: 95% Confidence interval
